# Quantum semi-supervised generative adversarial network for enhanced data classification

**DOI:** 10.1038/s41598-021-98933-6

**Published:** 2021-10-04

**Authors:** Kouhei Nakaji, Naoki Yamamoto

**Affiliations:** Department of Applied Physics and Physico-Informatics and Quantum Computing Center, Keio University, Hiyoshi 3-14-1, Kohoku, Yokohama 223-8522 Japan

**Keywords:** Quantum information, Information theory and computation

## Abstract

In this paper, we propose the quantum semi-supervised generative adversarial network (qSGAN). The system is composed of a quantum generator and a classical discriminator/classifier (D/C). The goal is to train both the generator and the D/C, so that the latter may get a high classification accuracy for a given dataset. Hence the qSGAN needs neither any data loading nor to generate a pure quantum state, implying that qSGAN is much easier to implement than many existing quantum algorithms. Also the generator can serve as a stronger adversary than a classical one thanks to its rich expressibility, and it is expected to be robust against noise. These advantages are demonstrated in a numerical simulation.

## Introduction

We are witnessing active challenges to develop several type of enhanced machine learning schemes via quantum computing, i.e., the research field of quantum machine learning^1–7^. In particular, based on the possible higher expressibility of quantum circuits over the classical one, which has been proven theoretically and demonstrated experimentally^8–12^, the quantum machine learning is expected to provide alternative subroutines to improve the performance. For instance, the quantum classifier^3,4,6,7^ is a quantum circuit that may be able to classify the input classical data with higher classification accuracy than conventional classical schemes such as the support vector machine. Note that many of those quantum machine learning applications need additional quantum operations or QRAM^13^ to load the classical data into the quantum circuit, which eventually may vanish the possible quantum advantage.

The machine learning scheme focused in this paper is the generative adversarial network (GAN)^14^. GAN is originally proposed in^14^ as a method for training generative models. In general, GAN consists of two adversarial components, typically a generator (generating a fake data) and a discriminator (discriminating real or fake data), and their adversarial training yields an outperforming system over the one trained solely. GAN has been successfully applied in the area of image processing and computer vision, including super resolution sensing^15–19^, image synthesis^20–27^, texture analysis^28–30^, object recognition^31–33^, and video^34–38^. Some works also proposed the application of GAN in other fields such as natural language processing^39–42^, chemistry^43–45^, and biology^46,47^; see^48,49^ for a recent review. GAN is also a topic actively studied in the quantum machine learning regime^50–64^. For example, Refs.^50,51^ provide a method to train the quantum generative model by using GAN, where both the discriminator and the generator are quantum systems. Also^52^ provides a method to synthesize a quantum generative model for discrete dataset, in the GAN framework. A useful application of GAN was proposed in^53^; their GAN consists of a quantum generator and a classical discriminator, and via the adversarial training the generator acquires the ability to generate the quantum state corresponding to a target probability distribution, which is then sent to another quantum circuit running a Grover-type algorithm (more precisely, the amplitude estimation algorithm for Monte Carlo simulation). Note that, however, this means that the generator is required to produce a target pure quantum state.

GAN also performs well in semi-supervised learning (SSL). SSL is a machine learning method that utilizes a dataset that contains both labeled and unlabeled samples as the training data^65–80^. By using those data, a prediction function $f_{\theta }$ with trainable parameters $\theta$ is optimized for predicting the label of a new sample better than the supervised learning that uses only labeled data, in the following sense. That is, the result of SSL tends to become robust compared to that of the supervised learning, because the unlabeled data corresponds to the underlying data distribution itself, the information of which effectively contributes to obtain a better decision boundary of the classifier. The nature of SSL that leverages unlabeled data to obtain a better performance is preferable especially when obtaining labeled data is costly. The idea of applying GAN to SSL was originally proposed in Ref.^73^ and there have been various studies^74–80^ since then. As in the case of standard GAN, the GAN in semi-supervised learning (SGAN) consists of a generator and a discriminator, but the role of the discriminator is to discriminate real/fake of the input data plus to estimate its label; that is, the goal of SGAN is to train the discriminator so that it becomes a good classifier, rather than realizing a high-quality generator. Notably, SGAN achieves competitive results in various SSL tasks^73–80^ (see also Section 3.5.1 in^48^). This is because the samples generated by the generator complement the training data so that the discriminator can find a better decision boundary^79^; hence, it is preferable that the generator has a rich expressibility power to effectively complement the training data.

In this paper, we propose the *quantum semi-supervised GAN (qSGAN)*. Our qSGAN consists of a quantum generator and a classical discriminator, like the case of^53^, but the main goal is to train the classical discriminator rather than the quantum generator. In other words, the quantum system is used to train the classical system. Therefore, our quantum generator needs neither any data loading nor to generate a pure quantum state. This means that qSGAN is much easier to implement than many existing quantum algorithms and may have a noise-tolerant property. Also the generator can serve as a stronger adversary than a classical one thanks to its rich expressibility, which may lead to a better classifier than the case using the classical SGAN. These advantages are demonstrated in a numerical simulation. In particular, we show that a small size qSGAN (32 parameters) successfully obtains a classifier that achieves almost the same classification accuracy as that obtained via a deep classical SGAN (3688 parameters); the former needs only 40 learning iterations while the latter does 400. Therefore qSGAN will pave the way to new applications of quantum computer, which might work even on a noisy quantum device.

We summarize our contributions as follows:

- We formulate the framework of qSGAN that utilizes the quantum generator, in such a way that the quantum generator needs neither any data loading nor to generate a pure quantum state.
- We demonstrate that, in a specific SSL problem, the quantum generator with higher expressibility leads to a classical classifier achieving a better classification accuracy. We also show that a small-size qSGAN can achieve almost the same classification accuracy as that obtained via a deep classical SGAN with much more parameters.
- We demonstrate the noise tolerant property of qSGAN, which implies that qSGAN is suitable for the execution even on a noisy quantum device.

## Results

### Algorithm of qSGAN

First let us recall the idea of standard GAN. GAN consists of a generator and a discriminator. The generator transforms a set of random seeds to samples (fake data). The discriminator receives either a real data from the data source or a fake data from the generator. Then the discriminator is trained so that it correctly classifies the received data into real or fake exclusively. Also the generator is trained so that its output (i.e., the fake data) are classified into real by the discriminator. Namely, the generator tries to fool the discriminator, and the discriminator tries to detect whether the received data is fake or real. If the training is successfully finished, then the generator acquires the ability to produce samples governed by a probability distribution that resembles the original distribution producing the real data. In what follows we describe the algorithm of qSGAN, based on the original proposal^75^.

#### Source of the real data

Suppose that we have $N_\text{B}$ data batches, and each batch contains $\ell$ labeled and $m-\ell$ unlabeled data. We write the set of labeled data in the *a*-th batch as $\{(\boldsymbol{x}^{a,1}_\text{L}, y^{a,1}), (\boldsymbol{x}^{a,2}_\text{L}, y^{a,2}),\ldots , (\boldsymbol{x}^{a,\ell }_\text{L}, y^{a,\ell })\}$ and that of unlabeled data as $\{\boldsymbol{x}^{a,1}_\text{UL}, \boldsymbol{x}^{a,2}_\text{UL}, \ldots , \boldsymbol{x}^{a, m-\ell }_\text{UL}\}$, where $y^{a, i}$ is the label of the *i*-th labeled data in the *a*-th batch. We summarize $\boldsymbol{x}^{}_\text{L}$ and $\boldsymbol{x}^{}_\text{UL}$ into a single data vector as

1 $$\begin{aligned} \boldsymbol{x}^{a,i}_\text{data} = {\left\{ \begin{array}{ll} \boldsymbol{x}^{a,i}_\text{L} &{} 1\le i \le \ell \\ \boldsymbol{x}^{a,i-\ell }_\text{UL} &{} \ell +1 \le i \le m \end{array}\right. }. \end{aligned}$$

The label data $y^{a,i}$ takes one of the values of $\{1, \ldots , c\}$, where *c* is the number of classes.

#### Generator

The generator is a quantum circuit composed of qubits, which prepares the quantum state $|\psi \rangle =U(\varvec{\theta })|0\rangle$, where $|0\rangle$ denotes the initial state of the circuit and $U(\varvec{\theta })$ is the unitary matrix corresponding to the parametrized quantum circuit with parameters $\varvec{\theta }$. The circuit outputs a fake data $\boldsymbol{x}^{}_\text{fake}$ as a result of the measurement of $|\psi \rangle$ in the computational basis. That is, $\boldsymbol{x}^{}_\text{fake}$ appears with probability $\mathbf{P}(\boldsymbol{x}^{}_\text{fake})=\left| \left\langle \boldsymbol{x}^{}_\text{fake}\left| U(\varvec{\theta })\right| 0\right\rangle \right| ^2$. Note that the stochasticity of the generator comes from this quantum intrinsic property, rather than the added random seeds like the classical GAN.

#### Discriminator/classifier

In the qSGAN framework, the discriminator $D(\boldsymbol{x})$, which judges real or fake for the received data $\boldsymbol{x}$, has an additional function, the label classifier $C(\boldsymbol{x})$. Hence, following Ref.^75^, we call this classical system simply the D/C. A more precise description of those functions is as follows. First, $D(\boldsymbol{x})$ represents the likelihood that the received $\boldsymbol{x}$ came from the real data source. Next, $C(\boldsymbol{x})$ is a vector whose dimension is $c+1$; the integer *j* ($1\le j \le c$) represents the likelihood that $\boldsymbol{x}$ belongs to the *j*-th class, and the last one is the likelihood that $\boldsymbol{x}$ is a fake data. In this paper, we use a double-headed classical neural network to implement $C(\boldsymbol{x})$ and $D(\boldsymbol{x})$;

2 $$\begin{aligned} C(\boldsymbol{x}) = g(f(\boldsymbol{x})), ~~~ D(\boldsymbol{x}) = k(f(\boldsymbol{x})). \end{aligned}$$

*f* is the function of the network shared by both $C(\boldsymbol{x})$ and $D(\boldsymbol{x})$, while *g* and *k* are the functions corresponding to the final layer; see the left of the Fig. 2.

#### Training rule

In the training process in each batch, *m* real data are loaded from the data source, and *m* fake data are generated by the generator, which are sent to the D/C. Then the D/C assigns a label to those data via $C(\boldsymbol{x})$ and also judges real/fake via $D(\boldsymbol{x})$. Based on those results, the parameters of the generator and the D/C are updated, according to the following rule.

First, the generator is updated so that the generated fake data are classified into real by the discriminator. More specifically, we minimize the following cost function $L_G$ to update the parameters $\varvec{\theta }$:

3 $$\begin{aligned} L_{G}= \mathbf{E}_{\boldsymbol{x} \sim \text{Generator}}(-\log D(\boldsymbol{x})) = \sum _{\boldsymbol{x}}\left( -\log D(\boldsymbol{x})\right) \left| \left\langle \boldsymbol{x}\left| U(\varvec{\theta })\right| 0\right\rangle \right| ^2 \simeq - \frac{1}{m} \sum _{i=1}^m \log \left( D(\boldsymbol{x}^{i}_\text{fake})\right) . \end{aligned}$$

In the above equation $\left| \left\langle \boldsymbol{x}\left| U(\varvec{\theta })\right| 0\right\rangle \right| ^2$ is the probability to have $\boldsymbol{x}$ when measuring the output state $U(\varvec{\theta })|0\rangle$ in the computational basis; hence the rightmost side of Eq. (3) follows from the law of large numbers, where $\boldsymbol{x}^{i}_\text{fake}$ is the *i*-th fake data obtained by measuring $U(\varvec{\theta })|0 \rangle$. Roughly speaking, minimizing $L_G$ corresponds to maximizing $D(\boldsymbol{x}^{i}_\text{fake})$, i.e., the likelihood that $\boldsymbol{x}^{i}_\text{fake}$ came from the real data source. In this paper, we take a quantum circuit where each parameter element $\theta _q$ is embedded into a single qubit gate in the form $U_q = \exp \left( - i \theta _q A_q /2 \right)$ with $A_q$ the single qubit operator satisfying $A_q^2=\mathbf{1}$. In this case, the gradient of $L_G$ with respect to $\theta _q$ boils down to the derivative $\partial \langle 0| U_q^\dagger W U_q |0\rangle /\partial \theta _q$ with *W* a Hermitian matrix which does not contain $\theta _q$. As a result, the gradient can be calculated in terms of the parameter-shifted circuits as follows^81^:

4 $$\begin{aligned} \frac{\partial L_G}{\partial \theta _{q}}&= \frac{1}{2}\sum _{\boldsymbol{x}}\left( -\log D(\boldsymbol{x})\right) \left( \left| \left\langle \boldsymbol{x}\left| U_{q +}(\varvec{\theta })\right| 0\right\rangle \right| ^2 - \left| \left\langle \boldsymbol{x}\left| U_{q -}(\varvec{\theta })\right| 0 \right\rangle \right| ^2\right) . \end{aligned}$$

where $U_{q\pm }(\varvec{\theta }) = U(\varvec{\theta } + \frac{\pi }{2} \boldsymbol{e}_q)$ is the parameter-shifted circuit, with $\boldsymbol{e}_q=[0, \ldots , 1, \ldots , 0]$ (only the *q*-th component is 1); that is,

5 $$\begin{aligned} U_{q \pm }(\boldsymbol{\theta })&=U_{q \pm }(\{\theta _1, \ldots , \theta _{q-1}, \theta _q, \theta _{q+1}, \ldots , \theta _n\}) \\&= U(\{\theta _1, \ldots , \theta _{q-1}, \theta _q\pm \pi /2, \theta _{q+1}, \ldots , \theta _n\}). \end{aligned}$$

Note now that $\left| \left\langle \boldsymbol{x}\left| U_{q +}(\boldsymbol{\theta })\right| 0\right\rangle \right| ^2$ and $\left| \left\langle \boldsymbol{x}\left| U_{q -}(\boldsymbol{\theta })\right| 0\right\rangle \right| ^2$ are the probability to have $\boldsymbol{x}$ when measuring the output states $U_{q +}(\boldsymbol{\theta })|0\rangle$ and $U_{q -}(\boldsymbol{\theta })|0\rangle$, respectively. Therefore, again from the law of large numbers, we get an unbiased estimator of $\partial L_G/\partial \theta _{q}$ as

6 $$\begin{aligned} \frac{\partial L_G}{\partial \theta _{q}} \simeq \frac{1}{2m}\sum _{i=1}^m \left[ -\log D(\boldsymbol{x}^{(q+)i}_\text{fake}) + \log D(\boldsymbol{x}^{(q-)i}_\text{fake})\right] , \end{aligned}$$

where $\boldsymbol{x}^{(q\pm ) i}_\text{fake}, (i=1, \ldots , m)$ are the fake data obtained by measuring $U_{q \pm }(\boldsymbol{\theta })| 0 \rangle$. This gradient descent vector is used to construct an optimizer for minimizing $L_G$.

Second, the D/C is trained so that it makes a correct real/fake judgement on the received data by $D(\boldsymbol{x})$ and, in addition, classifies them into the true class by $C(\boldsymbol{x})$. Hence, the parameters of the classical neural network are updated by minimizing the following cost function $L_{D/C}$:

7 $$\begin{aligned} L_{D/C}&= (L_{D} + L_{C})/2, \\ L_{D}&= \mathbf{E}_{\boldsymbol{x} \sim \text{Generator}}[-\log (1- D(\boldsymbol{x}))] + \mathbf{E}_{\boldsymbol{x} \sim \text{Source}}[-\log (D(\boldsymbol{x}))] \simeq -\frac{1}{m} \sum _{i=1}^m\left( \log \left( 1-D(\boldsymbol{x}^{i}_\text{fake})\right) +\log D(\boldsymbol{x}^{a,i}_\text{data})\right) , \\ L_{C}&= \mathbf{E}_{\boldsymbol{x} \sim \text{Generator}}[h(c+1, C(\boldsymbol{x}))] + \mathbf{E}_{\boldsymbol{x} \sim \text{Labeled~Source}}[h(y^i, C(\boldsymbol{x}))] \simeq \frac{1}{m}\sum _{i=1}^m h(c+1, C(\boldsymbol{x}^{i}_\text{fake})) + \frac{1}{\ell }\sum _{i=1}^{\ell } h(y^i, C(\boldsymbol{x}^{a,i}_\text{L})). \end{aligned}$$

Here $h(y, \boldsymbol{z}^{})$ is the cross entropy of the discrete distribution $q(i) = \exp (z_i)/(\sum ^{d}_{j=1}\exp (z_j))$ ($i=1,2,\ldots , d$) relative to the distribution $p(i) = \delta _{iy}$, defined as

8 $$\begin{aligned} h(y, \boldsymbol{z}^{}) = - z_y + \log \left( \sum _{j=1}^{d}\exp (z_{j})\right) , \end{aligned}$$

where $z_j$ is the *j*-th element of the vector $\boldsymbol{z}^{}$ and *d* is the dimension of $\boldsymbol{z}^{}$. As in the case of the generator, we use the gradient descent vector of $L_{D/C}$ to update the neural network parameters. In each iteration, the parameters are updated for all the batches. At the end of the training process with sufficiently large number of iterations, the trained D/C is obtained. The overall algorithm is summarized in Algorithm 1 with $N_\text{iter}$ the number of iteration.

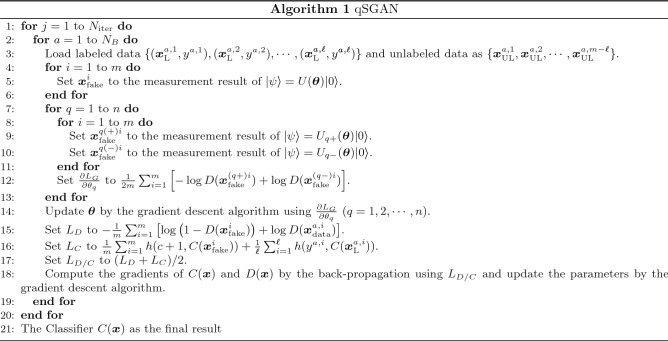



### Numerical demonstration

Here we demonstrate the performance of the proposed qSGAN method by a numerical simulation. In particular, we will see that a quantum generator with higher expressibility leads to a better classifier, after successful training. Also the resulting classification accuracy is comparable to that achieved when using a standard classical neural network generator.

#### Problem setting and result

**Figure 1:**
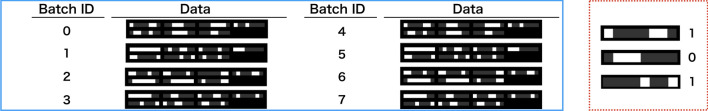
Left (enclosed by the blue rectangular): the dataset (= eight batches) used in the numerical simulation. Right (enclosed by the red dotted rectangular): examples of images and their labels.

The source of real data used in this simulation is a set of $1\times 8$ pixel images, shown in Fig. 1. Each pixel takes the value of 0 (black) or 1 (white). Also the label ‘0’ or ‘1’ is assigned to each image (hence $c=2$) according to the following rule; if white pixels in an image are all connected or there is only one white pixel in an image, then that image is labeled as ‘0’; If white pixels in an image are separated into two disconnected parts, then the image is labeled as ‘1’. The number of images with label ‘0’ and those with label ‘1’ are both 28 (hence 56 images in total). The dataset is separated into eight batches, each containing $m=7$ images.

**Figure 2:**
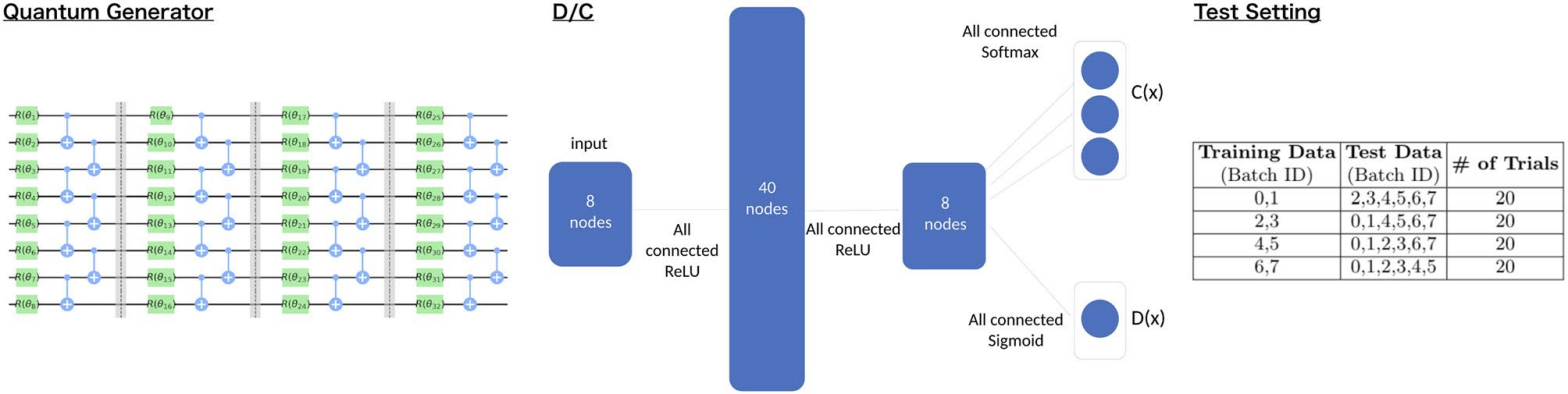
Left: the quantum circuit with four layers used in the simulation. Center: the D/C system, where the last layer functions as the classifier *C*(*x*) or the discriminator *D*(*x*). Right: the combination of the training/test dataset for the 4-fold cross validation.

As the quantum generator, we use a 8-qubits parametrized quantum circuit with single layer or four layers; the case of four layers is shown in the left of Fig. 2. Each layer is composed of parametrized single-qubit rotational gates $\exp (-i \theta _i \sigma _{a_i} /2)$ and CNOT gates that connect adjacent qubits; here $\theta _i$ is the *i*-th parameter and $\sigma _{a_i}$ is the Pauli operator ($a_i=x,y,z$). We randomly initialize all $\theta _i$ and $a_i$ at the beginning of each training. We run the numerical simulation on Qiskit QASM Simulator^82^.

As the D/C, we use a neural network with four layers, shown in the center of Fig. 2. The first three layers are shared by both the discriminator $D(\boldsymbol{x})$ and the classifier $C(\boldsymbol{x})$. The number of nodes in the first, second, and third layer are 8, 40, and 8, respectively; all nodes between the layers are fully connected, and we use ReLU as the activation functions. The last layer for the classifier has three nodes, corresponding to the likelihood of label “0” , label “1”, and fake classes; these nodes are fully connected to those of the third layer, and the softmax function is used as the activation function. The last layer of the discriminator has one node, simply giving the value of $D(\boldsymbol{x})$; this node is fully connected to the nodes of the third layer, and the sigmoid function ($\sigma (x) = 1/(1 + \exp (-x))$) is used as the activation function. We implement the neural networks by PyTorch^83^.

In each trial of the algorithm, we choose two of the eight batches as the training dataset (hence $N_B=2$) and the other six as the test dataset. We perform the 4-fold cross validation by changing the training and test dataset, as summarized in the right table of Fig. 2. For each training/test dataset, we execute 20 trials (80 trials totally). To demonstrate the semi-supervised learning, some of the labels in each batch are masked; recall that the number of labeled example in each batch is denoted by $\ell$, which takes $\ell =2$ or 5 in this simulation. As the gradient descent algorithm, we use Adam^84^, whose learning coefficient is set to 0.001 for the case of generator and 0.005 for the case of D/C.

**Figure 3:**
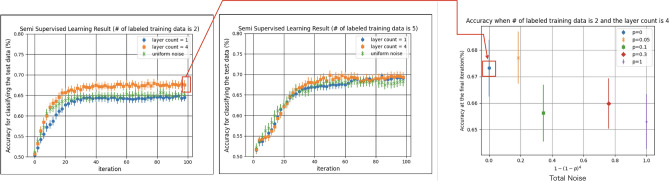
Left, center: classification accuracy of the classifier when using the quantum generator. The number of labeled data is $\ell =2$ (left) and $\ell =5$ (center). Right: classification accuracy of the classifier for $\ell = 2$ when using the four-layers quantum generator under the depolarization noise (9). The data points surrounded by the red rectangles are drawn with the same data.

The left and the center plots of Fig. 3 show the average classification accuracy for the test data versus the number of iteration, which are obtained as the average over 80 trials. The two subfigures are obtained with different number of labeled data, as $\ell =2$ (left) and $\ell =5$ (center). In each subfigure, three cases are shown, depending on the type of generator; the quantum generator with one layer (blue) and that with four layers (orange); also as a reference, the case of uniform-noise generator (green) that randomly generates 8-bit data with equal probability, which is not updated while training, is presented. The error bar represents the standard deviation of the average classification accuracy.

We see that, when only a few labeled data is available ($\ell =2$), the quantum generator with four layers results in the highest classification accuracy, which implies that the quantum generator with bigger expressibility contributes to the higher accuracy by effectively generating samples to train the classical D/C. This desirable property is supported by another numerical experiment with a larger dimensional dataset composed of $1 \times 16$ pixel images, showing that the generator with bigger number of layers (hence bigger expressibility) contributes to the better classification; see Supplemental Information 1. On the other hand, in the case where five of eight image data in each batch are labeled ($\ell =5$), the three generators achieve almost the same accuracy. This might be because, in this case, all the generators fail to generate more valuable dataset than the set of labeled real data, for effectively training the D/C. This observation is supported by the fact that the untrained uniform-noise generator, which of course is not related to the real dataset, achieves almost the same classification accuracy. Therefore, we expect that the quantum generator is useful when the number of labeled data is limited.

In this numerical simulation, we obtained the best classification accuracy when the constructed classical sample distribution corresponding to the output of the quantum generator does not match the distribution producing the real dataset, as predicted in^79^. In addition, we found that the cost for the quantum generator, $L_G$, is larger than that for the classical D/C, $L_{D/C}$, when the best accuracy is reached. These facts are favorable for the current noisy quantum devices that cannot be effectively trained due to the noise. Hence the next topic is to study how much the noise affects on the quantum generator and accordingly the classification accuracy.

#### Noisy qSGAN

We examine the case where a noise channel is applied between every layers of the quantum generator. In particular we assume the depolarizing channel:

9 $$\begin{aligned} \mathcal {E}(\rho ) = (1-p)\rho + p \frac{I}{2^n}, \end{aligned}$$

where $\rho$ is a density matrix, *I* is the identity matrix, *n* is the number of qubits, and *p* is a noise parameter. In this density matrix representation, the ideal unitary gate operation is expressed as $\mathcal {U}_i(\rho )=U_i \rho \, U_i^\dagger$, where $U_i$ is the *i*-th layer unitary matrix. Then, the output density matrix of the four-layers quantum circuit under the above depolarizing noise is given by

10 $$\begin{aligned} \rho _\text{out}& =\mathcal {E} \circ \mathcal {U}_4 \circ \mathcal {E} \circ \mathcal {U}_3 \circ \mathcal {E} \circ \mathcal {U}_2 \circ \mathcal {E} \circ \mathcal {U}_1 (|0\rangle \langle 0|) \\& =\mathcal {E} \circ \mathcal {U}_4 \circ \mathcal {E} \circ \mathcal {U}_3 \circ \mathcal {E} \circ \mathcal {U}_2 \left( (1-p) U_1|0\rangle \langle 0|U_1^{\dagger } + p\frac{I}{2^n} \right) \\& = \cdots = (1-p)^4 \, U_4 U_3 U_2 U_1 |0\rangle \langle 0| U_1^{\dagger }U_2^{\dagger }U_3^{\dagger }U_4^{\dagger } +\left( 1-(1-p)^4\right) \frac{I}{2^n}, \end{aligned}$$

where for instance, $\mathcal {E}\circ \mathcal {U}_i(\cdot )$ denotes the composite function $\mathcal {E}\left( \mathcal {U}_i(\cdot )\right)$ of $\mathcal {E}(\cdot )$ and $\mathcal {U}_i(\cdot )$. The samples are generated by measuring $\rho _\text{out}$. Other than the presence of noise, the simulation setting are the same as the noiseless case. We examine the case when only two of eight image data in each batch are labeled ($\ell =2$).

The resulting classification accuracy achieved via the quantum generator under the depolarization noise is shown in the right of Fig. 3, with several values of noise strength *p*. The horizontal axis represents the magnitude of total noise, i.e., the coefficient of the second term in Eq. (10), while the vertical axis represents the average classification accuracy at the final (= 100-th) iteration step. The error bar is the standard deviation of the average accuracy. The result is that, as discussed before, the classification accuracy does not become worse than the noiseless case, as long as the depolarization noise for each layer of the quantum generator is suppressed to some level ($p=0.05$). This demonstrates the second advantage of the proposed qSGAN described in Introduction; that is, the quantum generator in our qSGAN framework does not need to generate a pure quantum state.

To further validate the noise-tolerant property of qSGAN under the noise, we consider typical classification metrics in addition to the accuracy, in the case of $\ell =2$ for various noise level. The additional metrics are the precision and the recall, for the connected (label $=0$) and the disconnected (label $=1$) samples. The precision and the recall for the connected samples, which are respectively denoted by *P*(0) and *R*(0), are defined as

11 $$\begin{aligned} P(0) = \frac{True(0)}{True(0) + False(0)}, \qquad R(0) = \frac{True(0)}{True(0) + False(1)}, \end{aligned}$$

where $True(j)\ (j=0,1)$ is the number of samples correctly classified as *j* and $False(j)\ (j=0,1)$ is the number of samples incorrectly classified as *j*, at the final iteration step. Similarly, the precision and the recall for the disconnected samples, which are denoted by *P*(1) and *R*(1), are defined as

12 $$\begin{aligned} P(1) = \frac{True(1)}{True(1) + False(1)}, \qquad R(1) = \frac{True(1)}{True(1) + False(0)}. \end{aligned}$$

As in the case of accuracy, higher precision and recall mean that we obtain a better classifier.

**Figure 4:**
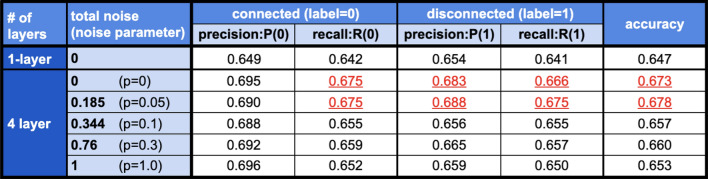
Classification metrics of qSGAN, in the case of $\ell =2$ for various depolarization noise level *p*. The total noise in the quantum generator with four layers is computed as $1-(1-p)^4$. The two highest values of each metric that are statistically significantly higher than the others are highlighted in red and underlined.

Figure 4 shows the averaged values of precision, recall, and accuracy, over 80 trials. The two highest values of each metric that are statistically significantly higher than the others are highlighted in red and underlined. Notably, the conclusion deduced from the values of *R*(0), *P*(1), and *R*(1) is the same as that for the case of accuracy; that is, the classification performance does not become worse than the noiseless case, as long as the noise parameter is suppressed below $p=0.05$. Readers may notice that *P*(0) does not become worse even if strong ($p\ge 0.1$) depolarization noise is appended while the other metrics become worse. This phenomenon seems to be unique to this dataset, but further study is needed to see the reason.

#### Comparison with a classical neural network generator

**Figure 5:**
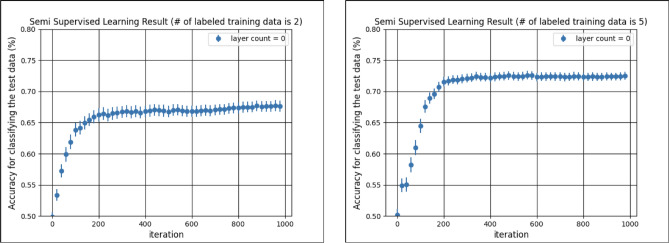
Classification accuracy of the classifier when using the five-layers classical neural network generator. The number of labeled data is $\ell =2$ (left) and $\ell =5$ (right).

Finally, we compare the performance of the proposed qSGAN to the fully classical case where the generator is given by a five-layers classical neural network. The input to the generator is the 1-dimensional normal Gaussian noise with zero mean and unit variance. The second, third, and fourth layer are composed of 40 nodes, and the fifth (= final) layer has 8 nodes. The nodes between the layers are fully connected and ReLU is used as the activation function. The output sample is obtained by transforming the values of the nodes at the final layer by the sigmoid function ($\sigma (x) = 1/(1 + e^{-x})$). We use the same D/C used in the quantum case. As the gradient descent algorithm, we use Adam, whose learning coefficients are set to 0.001 for both the generator and the D/C. Figure 5 shows the classification accuracy for the test data over the number of iteration, which are obtained as the average over 80 trials when using the classical neural network generator. The two subfigures are obtained with different number of labeled data, $\ell =2$ and $\ell =5$.

**Figure 6:**
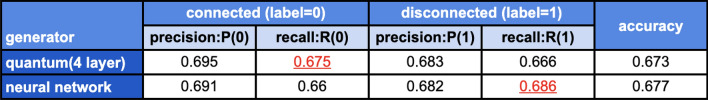
Classification metrics of qSGAN with four layers quantum generator and the classical SGAN with five layers neural network generator. The number of labeled data is $\ell =2$. The highlighted value indicates that it is statistically and significantly higher than the complement in each metric.

The result is that, for the case $\ell =2$, the classifier aided by the classical neural network generator achieves the classification accuracy about 67$\%$, which is comparable to that of the four-layers quantum generator shown in the left subfigure of Fig. 3. This competitive performance of these quantum generator and the classical neural network generator can also be observed when evaluating the precision and the recall in Fig. 6; the highlighted value indicates that it is statistically and significantly higher than the complement in each metric. The notable point is that the number of parameters of the classical and quantum generators are 3688 and 32, respectively. Hence, naively, the quantum generator has a rich expressibility power comparable to the classical one even with much fewer parameters. This means that the training of the quantum generator is easier than the classical one, which is actually shown in Figs. 3 and 5; about 40 iterations is enough to reach the accuracy 67$\%$ for the former case, while the latter requires roughly 400 iterations to reach the same accuracy. More importantly, this result implies that a bigger quantum generator with tractable number of parameters could have a potential to work even for some problems that are intractable via any classical one due to the explosion of the number of parameters.

## Discussion

In this paper, we developed qSGAN that performs a semi-supervised learning task by GAN composed of the quantum generator and the classical discriminator/classifier. This system has the following clear merits. That is, the quantum generator needs neither data loading nor to generate a pure quantum state; rather its main role is to train the classical classifier, as a possibly stronger adversary than a classical one. The numerical experiment using the connected/disconnected image dataset shows that the rich expressibility of the quantum generator contributes to achieve the classification accuracy as high as that obtained when using the deep classical neural network generator (hence with much more parameters involved there). Also, we exemplified the noise-tolerant property of qSGAN under the depolarization noise, which is also a preferable feature for implementing qSGAN on a current noisy quantum device.

To evaluate the real applicability of qSGAN beyond the proof-of-concept demonstration provided in this paper, it is important to compare qSGAN with several classical SSL algorithms^65–72,74–80^, in some standard benchmark problems. As a typical problem, let us consider the binary MNIST dataset (28 $\times$ 28 pixels, black/white image) studied in^74^, in which several classical algorithms achieving at most 90$\%$ accuracy were presented. For this problem, the quantum generator needs 784 qubits, which is expected to have some superiority over classical means, for the following reason. That is, a quantum circuit may have a bigger expressibility power than a similar-sized classical neural network, while a generator with bigger expressibility can well complement the dataset for training and thereby lead to a higher-performance classifier. Actually a big-size quantum circuit is difficult to simulate via any classical computer, and thus a quantum generator with hundreds of qubits may lead to a better performance in the SGAN formulation for this benchmark MNIST problem. Of course such device will be noisy, but, as shown in “Noisy qSGAN” section, qSGAN has some robustness against such noise and thus expected to work even in the absence of noise-tolerance. Another issue is the difficulty for training the parametrized quantum circuit on the 784 qubits device (known as the barren plateau issue), but the point of SGAN is that the generator needs not perform highly precisely; in fact, as shown in^79^, sometimes a low-performance generator leads to a high-performance classifier that even achieves almost the best performance in the SSL framework. Because the 784-qubits device is not available now and also classical simulation for this system is impossible, we studied the 8 qubits example as a proof-of-concept demonstration. Nonetheless, the additional 16 qubits example given in Supplemental Information 1 shows that the algorithm still performs well, implying that qSGAN will work for bigger-size problems as well, including the benchmark MNIST problem.

## Supplementary Information

Supplementary Information.

## Data Availability

The data that support the findings of this study are available from the corresponding author upon reasonable request.
