# Supplementary material for: Quantum semi-supervised generative adversarial network for enhanced data classification
Source: Sci Rep. 2021 Oct 4;11:19649. doi: 10.1038/s41598-021-98933-6 (PMC8490428; doi:10.1038/s41598-021-98933-6)
Supplement: Supplementary file 1 — Supplementary Information. [file 41598_2021_98933_MOESM1_ESM.pdf]

# Supplemental Information

## NUMERICAL EXPERIMENT USING 16-DIMENSIONAL DATASET

We demonstrate the performance of proposed qSGAN using dataset with larger dimension. The source of real data used in this simulation is a set of $1 \times 16$ pixel images, shown in Figure S1. As the experiment in the main text, each pixel takes the value of 0 (black) or 1 (white) and the label '0' or '1' is assigned to each image according to the same rule as the one in main text. The number of images with label '0' and those with label '1' are both 120 (hence 240 images in total). The dataset is separated into eight batches, each containing $m = 30$ images.

As the quantum generator, we use a 16-qubits parametrized quantum circuit with single layer, four layers, or eight layers. Each layer is composed of parametrized single-qubit rotational gates $\exp(-i\theta_i\sigma_{a_i}/2)$ and CNOT gates that connect adjacent qubits. We randomly initialize all $\theta_i$ and $a_i$ at the beginning of each training. We run the numerical simulation on Qiskit QASM Simulator.

As the D/C, we use a similar neural network with four layers used in the main text. The first three layers are shared by both the discriminator $D(\boldsymbol{x})$ and the classifier $C(\boldsymbol{x})$. The number of nodes in the first, second, and third layer are 16, 40, and 16, respectively; all nodes between the layers are fully connected, and we use ReLU as the activation functions. The last layer for the classifier has three nodes, corresponding to the likelihood of label '0', label '1', and fake classes; these nodes are fully connected to those of the third layer, and the softmax function is used as the activation function. The last layer of the discriminator has one node, simply giving the value of $D(\boldsymbol{x})$; this node is fully connected to the nodes of the third layer, and the sigmoid function is used as the activation function. We implement the neural networks by PyTorch.

In each trial of the algorithm, we choose two of the eight batches as the training dataset (hence $N_B = 2$) and the other six as the test dataset. We perform the 4-fold cross validation by changing the training and test dataset. For each training/test dataset, we execute 10 trials (40 trials totally). To demonstrate the semi-supervised learning, some of the labels in each batch are masked; recall that the number of labeled example in each batch is denoted by $\ell$, which takes $\ell = 2$. As the gradient descent algorithm, we use Adam, whose learning coefficient is set to 0.005 both for the generator and the D/C.

Figure S2 shows the average classification accuracy for the test data versus the number of iteration, which are obtained as the average over 40 trials. In the figure, our four cases are shown, depending on the type of generator; the quantum generator with one layer (blue), that with four layer (orange) and that with eight layer (red); also as a reference, the case of uniform-noise generator (green) that randomly generates 16-bit data with equal probability, which is not updated while training, is presented. The error bars represent the standard deviation of the average classification accuracy.

We see that the more layer a quantum generator has, the higher classification accuracy is obtained. It is consistent with the result in the main text; the quantum generator with bigger expressibility contributes to the higher accuracy.

In Figure S3, we show the averages of precision and recall, which are defined in the main text as $P(0)$, $R(0)$, $P(1)$, and $R(1)$, for each generator. In the same figure, we show the average accuracy. The highest values of each metric that is statistically significantly higher than the others are highlighted in red and underlined.

Even with these metrics, the highest classification performance seems to be obtained when we use the generator with 8 layers; other than $R(0)$, the quantum generator with 8 layers achieves the highest values. Even though the quantum generator with one layer achieves the highest value of $R(0)$, that is because the resulting classifier $C(x)$ tends to classify any data as '0'; as a result, $R(0)$ and $R(1)$ becomes much worth than the ones when using the other generators. From these

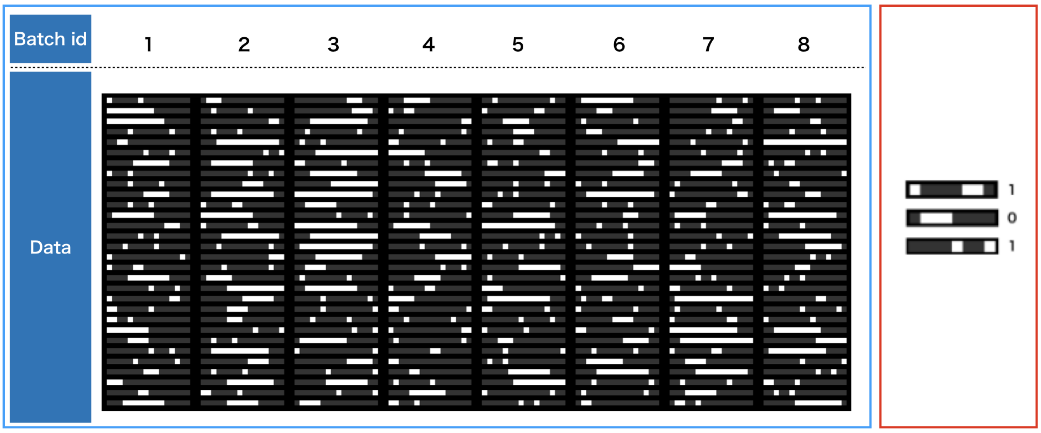


**Fig. S1.** Left (enclosed by the blue rectangular): The 16-dimensional dataset (=eight batches) used in the numerical simulation. Right (enclosed by the red dotted rectangular): Examples of images and their labels.

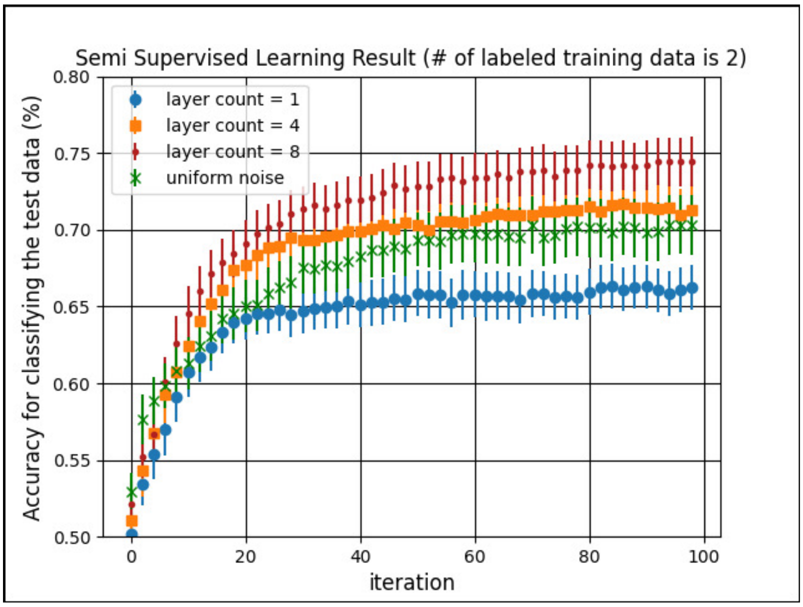


**Fig. S2.** Classification accuracy of the classifier when using the quantum generator depending on the type of generator; the quantum generator with one layer (blue), that with four layer (orange) and that with eight layer (red). Also as a reference, the case of uniform-noise generator (green) that randomly generates 16-bit data with equal probability, which is not updated while training, is presented. The number of labeled data is $\ell = 2$.

| generator | connected (label=0) | | disconnected (label=1) | | accuracy |
|---|---|---|---|---|---|
| | precision:P(0) | recall:R(0) | precision:P(1) | recall:R(1) | |
| quantum(1 layer) | 0.668 | 0.703 | 0.696 | 0.622 | 0.662 |
| quantum(4 layer) | 0.759 | 0.655 | 0.708 | 0.767 | 0.710 |
| quantum(8 layer) | 0.807 | 0.664 | 0.736 | 0.823 | 0.734 |
| uniform noise | 0.739 | 0.680 | 0.724 | 0.732 | 0.706 |

**Fig. S3.** Classification metrics when $\ell = 2$ with each layer of the quantum generator. The case of the uniform-noise generator is also presented. The highest value of each metric that is statistically significantly higher than the others are highlighted in red and underlined.

observations, it can be said that the best results are obtained when using the generator with the highest expressive power, even when using metrics other than the accuracy.
